# Scoping the Landscape of Deep Learning for Alzheimer’s Disease Stage Classification: Methods, Challenges, and Opportunities

**DOI:** 10.34133/bmef.0202

**Published:** 2025-11-27

**Authors:** Salleh Sonko, Mohamed Islam Houssam, Kossi Dodzi Bissadu, Brian O’Connor, Gahangir Hossain

**Affiliations:** ^1^ Department of Information Science, University of North Texas, Denton, TX, USA.; ^2^ Department of Electrical and Electronics Engineering, Islamic University of Technology, Gazipur–Dhaka, Bangladesh.; ^3^ Department of Data Science, University of North Texas, Denton, TX, USA.

## Abstract

Deep learning (DL) models have been widely applied for Alzheimer’s disease (AD) stage classification. This scoping review synthesizes recent research to evaluate current performance benchmarks, identify methodological limitations, and highlight translational barriers. DL has potential to augment diagnostic accuracy and accelerate early intervention in AD, but translation requires models that generalize across datasets and integrate into real-world clinical workflows. Following scoping review methodology, 18 peer-reviewed studies published between 2018 and 2024 were analyzed. We extracted dataset sources, preprocessing strategies, model architectures, performance metrics, and translational considerations. Most studies employed convolutional neural networks (CNNs) or transfer learning (TL) backbones with accuracies frequently reported above 90%. Comparative synthesis revealed that TL and custom CNNs achieved similar headline accuracies, with differences of less than one percentage point. Reported performance was highly sensitive to task framing (cross-sectional vs. progression) and dataset provenance, with curated subsets often yielding near-ceiling internal accuracies but limited generalizability. Only one study implemented true external validation, underscoring a critical translational gap. Cost-effectiveness was rarely discussed explicitly; however, several studies indicated that open datasets reduce financial barriers, while adapting pipelines for EMR, or multisite data entails substantial resource demands. DL for AD classification shows consistent high accuracy but limited robustness, with external validation and financial cost-effectiveness remaining underreported. Future progress depends on standardized evaluation protocols, explicit reporting of financial costs, and the development of clinically interpretable, workflow-integrated models.

## Introduction

Alzheimer’s disease (AD) is an irreversible, progressive neurodegenerative disorder that accounts for approximately 60% to 70% of global dementia cases [1,2]. AD causes brain tissue atrophy that leads to neuronal death, leading to loss and impairment of various cognitive functions, including reading, writing, speaking, and performing daily tasks [3]. In 2019, the Alzheimer’s Association reported that 5.8 million US citizens were affected by AD, including 200,000 under 65 years of age, 2.6 million 75 to 84 years of age, and 2.1 million 85 years of age or older [4]. AD is the seventh leading cause of death in the United States, surpassing breast and prostate cancer combined [5].

Advancements in understanding the biological mechanisms of AD reveal pathological brain changes, including the accumulation of beta-amyloid plaques and tau tangles. These changes normally begin 15 to 20 years before symptoms manifest, a phase referred to as preclinical AD [6]. Although the pathophysiology and risk factors for AD remain elusive [7], Dominy et al. [3] reported that family history, genetics, and environmental factors play a crucial role. Itzhaki [8] associates AD with herpes simplex virus type 1, while Braak and Braak [9] suggest that the onset of AD is age-related but not strictly age-dependent.

Currently, the diagnosis and progression monitoring of AD are based on the observation of clinical symptoms and medical history and conducting cognitive tests such as the Global Deterioration Scale [10], Clinical Dementia Rating [11], and Mini-Mental State Examination [12]. However, by the time symptoms appear, the disease has already progressed substantially , diminishing the effectiveness of treatment [13]. Consequently, an artificial intelligence-based method for clinically detecting AD or monitoring its progression between appointments would be valuable. This could aid in adjusting treatment plans, slowing down progression, or implementing preventive measures.

Deep learning (DL) techniques, with their ability to analyze complex data and recognize patterns, are revolutionizing the field of AD research. They have shown promising results in the identification of AD from neuroimaging data, offering a new avenue for understanding and treating this complex disease. As indicated by Ebrahimighahnavieh et al. [14], DL approaches have demonstrated effectiveness in diagnosing AD from neuroimaging data. The potential to assess pathophysiological changes on magnetic resonance imaging (MRI) scans holds the promise of uncovering new treatments for AD, instilling hope in patients, their families, and clinicians [15].

In contrast to conventional machine learning (ML) algorithms, DL techniques excel at evaluating medical images without requiring extensive preprocessing or feature engineering [16]. Furthermore, DL models prove effective in handling various challenges such as different time and space complexities, statistical data distribution, convergence, and overfitting—common issues encountered when using conventional ML models in AD diagnosis.

According to Nawaz et al. [17], AD is a multiclass problem. However, much of the research in this field concentrates on the construction of binary classifiers that determine whether an individual has AD or not. However, given the urgent need to detect clinical deterioration between medical appointments for AD patients, it becomes imperative to assess the necessity of treatment adjustments or the implementation of preventive measures, such as increased surveillance or the encouragement of participation in stimulating activities.

Our study offers a comprehensive review of DL models developed specifically to identify various stages of AD. We explore the data modalities utilized and the preprocessing techniques employed in these studies, including any data augmentation strategies implemented to mitigate issues such as class imbalance or the so-called curse of dimensionality using principal component analysis (PCA). Furthermore, we examine the diverse convolutional neural network (CNN) architectures covered in the included studies, along with the limitations reported and the future directions proposed. In addition, we synthesize findings across studies to compare the performance of transfer learning (TL) and custom CNN models, emphasizing how dataset provenance, class granularity, and task framing influence reported outcomes. Finally, we highlight the importance of addressing clinical translation challenges—such as limited external validation, workflow integration, and cost-effectiveness—to ensure these models can be effectively deployed in real-world healthcare settings.

## Methodology

**Fig. 1.**
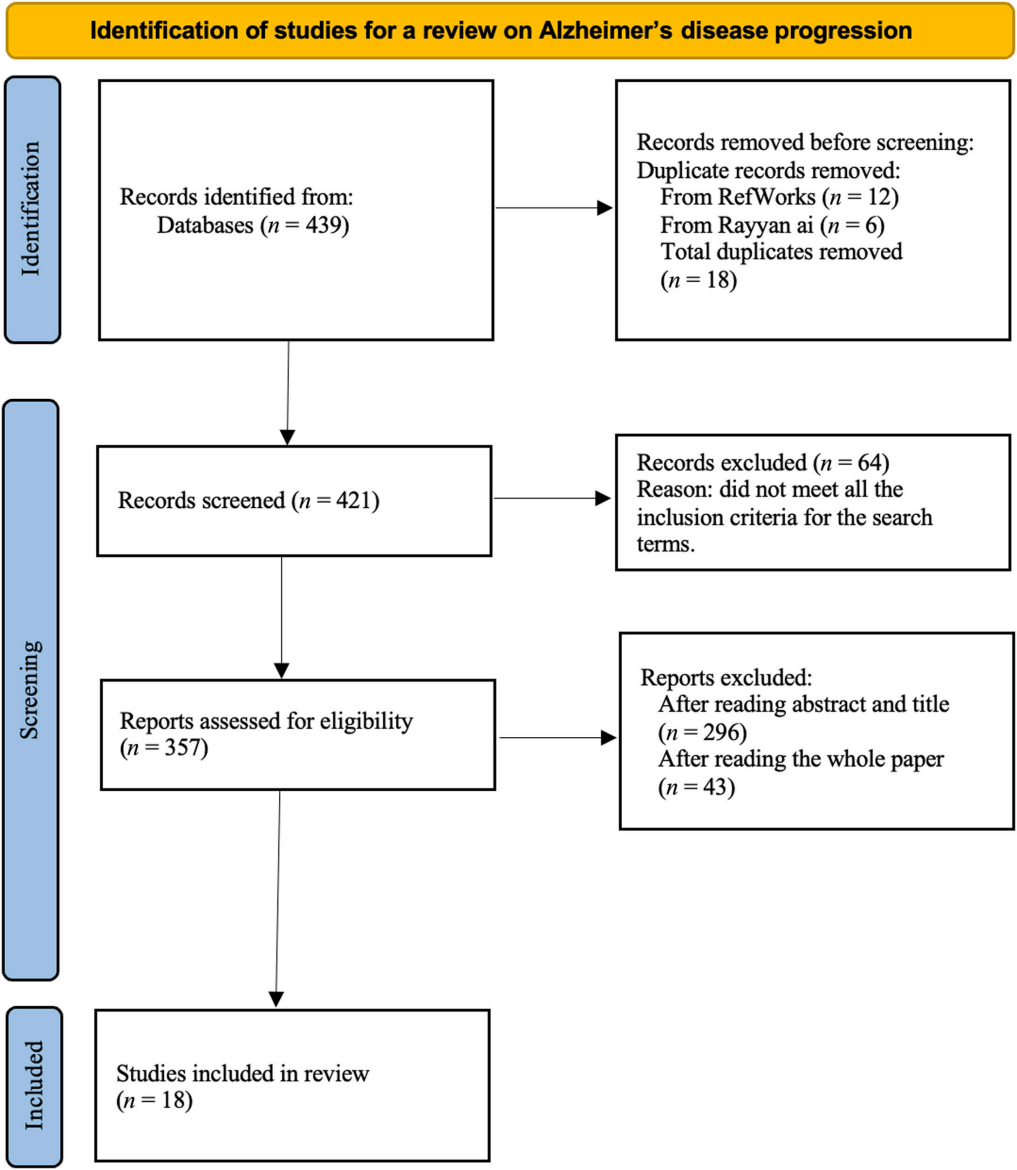
PRISMA-ScR flow diagram illustrating the identification, screening, eligibility, and inclusion process for studies on Alzheimer’s disease stage classification. The diagram follows the PRISMA 2020 extension for scoping reviews [18].

This scoping review investigates the use of DL techniques in identifying different stages of AD. The review adheres to the Preferred Reporting Items for Systematic Reviews and Meta-Analyses extension for Scoping Reviews (PRISMA-ScR) guidelines [18], ensuring a transparent and systematic approach to study identification, selection, and data extraction. The study selection process is illustrated in Fig. 1 and includes peer-reviewed research published between 2015 and 2025.

The inclusion period begins in 2015 to align with the emergence of DL applications in neuroimaging for AD research. While foundational work on DL in medical imaging predates this period, it was in 2015 that the field witnessed a marked increase in the application of CNNs and autoencoders to structural and functional neuroimaging data—especially MRI and positron emission tomography (PET). Notably, Payan and Montana [19] introduced a 3-dimensional CNN model for AD diagnosis using structural MRI (sMRI), representing one of the earliest implementations of DL in this context. Similarly, Suk et al. [20] applied stacked autoencoders for distinguishing between AD and mild cognitive impairment (MCI), demonstrating the viability of unsupervised DL techniques in neuroimaging-based classification tasks. These pioneering studies laid the groundwork for a surge in DL-based approaches to AD staging, making the 2015 to 2025 period a critical developmental window for the field.

We conducted a comprehensive search using PubMed, Web of Science, and Google Scholar. The search strategy incorporated relevant keywords and MeSH terms covering the disease (e.g., “Alzheimer’s Disease”, “dementia”, and “Alzheimer’s progression”), interventions (e.g., “deep learning” and “CNN”), and outcomes (e.g., “stage classification” and “progression prediction”). The results were filtered to include only English-language peer-reviewed journal and conference papers published in the past 10 years, yielding 439 records. All retrieved records were first imported into RefWorks to identify and remove duplicate papers. Additional deduplication and screening processes were conducted using Rayyan.ai, a web-based tool designed to facilitate systematic reviews. In total, 18 duplicates were identified—12 removed through RefWorks and an additional 6 via Rayyan.ai. Each duplicate was manually verified to ensure complete redundancy and accuracy. Following deduplication, 421 unique records remained for the screening phase.

An initial screening of titles and abstracts resulted in the exclusion of 64 studies that did not meet the predefined inclusion criteria. The majority of these exclusions were due to a focus on neurodegenerative conditions unrelated to AD, such as Parkinson’s disease.

Of the remaining 357 studies assessed for eligibility, 296 were excluded based on abstract and title review. These papers largely focused on binary classification tasks (e.g., distinguishing AD from normal control) or on MCI, which is considered the prodromal stage of AD and falls outside our scope of multistage AD progression analysis. An additional 43 studies were excluded after full-text review due to similar scope mismatch.

In total, 18 studies met all inclusion criteria and were included in the final synthesis. These studies involved human subjects with AD, applied DL methods to disease staging or progression prediction, and provided empirical findings published in English-language peer-reviewed outlets between 2015 and 2025.

## Results

### Publication trends in DL applications for AD stage identification

Research specifically targeting the identification of AD stages using DL techniques has only gained momentum in recent years. The earliest relevant publication in this area appeared in 2017, when Islam and Zhang [21] introduced a novel DL-based approach for classifying the stages of AD, marking a foundational step toward stage-specific disease modeling. During the initial years, however, scholarly interest in this domain remained limited—perhaps due to the prevailing perception that, given the incurable nature of AD, developing models to assess disease severity or progression would have limited immediate clinical benefit. As a result, early research efforts were primarily focused on detecting the onset of AD, especially during its prodromal phase, such as MCI.

By 2018, only one additional study had been published, and 2019 saw no new contributions. A shift began in 2020, with 3 studies published, suggesting an emerging recognition of the potential clinical value in identifying disease stages.

This growing interest was sustained modestly in 2021 with 2 publications and continued with 1 in 2022. A substantial increase occurred in 2023, with 9 studies published—representing the peak of scholarly attention within the observed timeframe. This surge likely reflects broader acknowledgment of DL’s potential in advancing clinical decision-making and patient management in the context of AD. However, the upward trend did not last; only one study was published in 2024, and as of mid-2025, no new studies have been recorded.

### Databases and data modalities

**Fig. 2.**
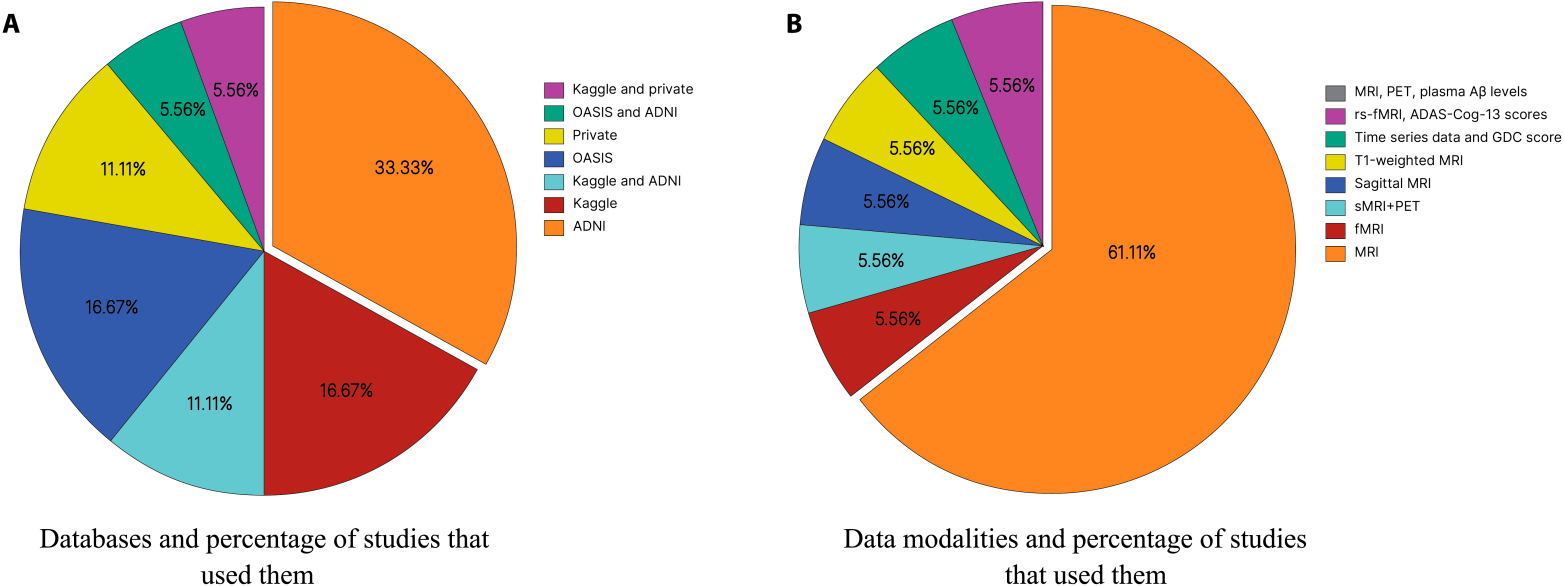
The (A) databases and (B) data modalities used in the surveyed studies (in percentage).

This review examines the sources of data and imaging modalities employed in the 18 studies focused on AD diagnosis and progression. As shown in Fig. 2, researchers predominantly relied on publicly available neuroimaging datasets, often supplemented with multimodal imaging approaches.

Among the databases, the Alzheimer’s Disease Neuroimaging Initiative (ADNI) was the most frequently used, appearing in 32.7% of the reviewed studies. Initiated in 2003 as a $60 million public–private partnership, ADNI aims to evaluate imaging, biological markers, and neuropsychological assessments to monitor the progression of MCI and early AD [22].

Following ADNI, both the Kaggle AD dataset and the Open Access Series of Imaging Studies (OASIS) were utilized in 16.8% of studies each. The Kaggle dataset provides preprocessed MRI scans suitable for ML applications [23], while OASIS is a well-curated neuroimaging database by researchers from the Howard Hughes Medical Institute at Harvard University, the Neuroinformatics Research Group at Washington University School of Medicine, and the Biomedical Informatics Research Network [24]. Several studies also employed combinations of databases—such as Kaggle + ADNI and OASIS + ADNI (10.9% each)—to enhance sample diversity. Smaller subsets of studies utilized private datasets or merged Kaggle with private sources (5.9% each). Figure 2A presents an overview of the databases utilized in the analyzed studies, expressed as percentages.

Regarding data modalities, horizontal MRI was the most prevalent, used in 59.2% of the reviewed studies. This strong preference may be attributed to horizontal MRI’s ability to provide high-resolution structural information of the brain in axial slices, which are particularly effective for visualizing key regions implicated in AD, such as the hippocampus, entorhinal cortex, and medial temporal lobes. These regions exhibit characteristic atrophy in the early stages of the disease, and their consistent appearance across axial slices facilitates reliable feature extraction for DL models. Although sagittal-plane MRIs, sMRI, functional MRI (fMRI), and so on are less frequently used, studies such as that of Puente-Castro et al. [25] have shown that particularly sagittal views can be equally effective for early AD detection; however, axial imaging remains the standard in most DL-based neuroimaging research.

Furthermore, horizontal MRI is widely available in public datasets, is standardized across institutions, and typically requires less preprocessing than other modalities—making it well-suited for training DL classifiers. In contrast, other modalities—including fMRI, sMRI combined with PET, sagittal MRI, T1-weighted MRI, time-series data with Global Deterioration Score, rs-fMRI combined with ADAS, and MRI with PET—each appeared in only 5.8% of the studies. Although these approaches offer complementary functional or multidimensional perspectives, they often demand more complex preprocessing, alignment, or multimodal fusion techniques, which may limit their accessibility or integration into scalable DL pipelines. While such modalities hold significant potential for future research, they remain underutilized in current Alzheimer’s classification efforts using DL. Figure 2B presents the percentages of each data modality employed in the reviewed studies.

### Preprocessing techniques employed in the included studies

#### Description of preprocessing techniques

The performances of artificial intelligence-based diagnostic systems, particularly those employing DL models, are significantly influenced by the effectiveness of preprocessing of input data. Preprocessing serves multiple crucial functions, including mitigating class imbalance, eliminating noise and artifacts, standardizing data formats, and optimizing feature representation. While most DL architectures may not require extensive preprocessing steps, the majority of studies reviewed in this work incorporated specific preprocessing procedures in their methodologies to enhance model reliability and accuracy.

Among the 18 studies analyzed, image resizing to a common input dimension emerged as the most frequently employed preprocessing technique, reported in 10 studies (56%). This form of standardization involves adjusting all images to a consistent size—commonly 224 *×* 224 *×* 3—to ensure compatibility with CNN architectures. Standardizing input dimensions is critical for enabling batch processing, reducing computational complexity, and maintaining consistency across training and validation stages, thereby enhancing model performance and stability [17,21,26–33].

To address the common issue of class imbalance—particularly prevalent in AD datasets—data augmentation techniques were utilized in 8 studies (44%). These techniques aim to synthetically increase the training dataset, thereby improving model generalizability and reducing overfitting. Common augmentation methods included geometric transformations such as cropping, flipping, rotation, and scaling. In some studies, more advanced augmentation approaches—such as image synthesis using generative adversarial networks (GANs) and Synthetic Minority Oversampling Techniques (SMOTE)—were employed to enrich the minority class and balance the dataset more effectively [21,26–28,31,33,34].

Filtering methods were employed in 3 studies (17%) to reduce noise and enhance image quality, thereby facilitating more accurate and reliable feature extraction. These techniques help suppress irrelevant variations and artifacts in the input data, which can otherwise degrade model performance. Examples from the reviewed studies include the use of guided filtering [34], bilateral filtering [27], and quantum matched filtering [35], each tailored to preserve important structural details while minimizing noise.

To address the so-called curse of dimensionality—a well-known challenge in ML and DL models, particularly when handling the high-dimensional feature spaces generated by CNNs—dimensionality reduction techniques were applied in 2 studies (11%). PCA, the most commonly used method in this context, was employed to project the data onto a lower-dimensional subspace while retaining the most informative features. This not only reduces computational complexity but also mitigates the risk of overfitting by eliminating redundant or less relevant features [35,36].

Finally, a category labeled as “Others” (6 studies, 33%) encompassed a range of specialized preprocessing strategies designed for neuroimaging data. These techniques included deskulling (removal of nonbrain tissues), tissue segmentation (differentiating gray matter, white matter, and cerebrospinal fluid), and template matching—procedures particularly relevant for enhancing the quality and anatomical accuracy of MRI data in brain imaging tasks [25,33,37–40].

A summary of the frequency and distribution of these preprocessing techniques is presented in Table 1.

**Table 1.** Preprocessing techniques used in the reviewed studies

Preprocessing technique	No. of studies (%)	References
Data standardization	10 (56%)	[17,21,26–33]
Data augmentation	8 (44%)	[21,26–28,31,33,34]
Filtering	3 (17%)	[27,34,35]
Dimensionality reduction (e.g., PCA)	2 (11%)	[35,36]
Other techniques (e.g., deskulling and segmentation)	6 (33%)	[25,33,37–40]

#### Detailed description of preprocessing techniques

Table 2 offers a detailed breakdown of the preprocessing techniques employed across the reviewed studies, providing a more granular view than the summary in Table 1. The techniques are grouped into 5 primary categories: data standardization (image resizing), data augmentation, filtering, dimensionality reduction, and other specialized operations tailored for neuroimaging data.

**Table 2.** Detailed breakdown of preprocessing techniques in the surveyed studies

Preprocessing technique (subtype)	No. of studies (%)	References
**Data standardization (image resize)**	10 (100%)	
224 × 224 × 3	3 (30%)	[30,32,33]
128 × 128 × 3	2 (20%)	[26,28]
176 × 176 × 3	1 (10%)	[27]
100 × 100 × 3	1 (10%)	[29]
112 × 112 × 3	1 (10%)	[31]
227 × 227 × 3	1 (10%)	[17]
229 × 229 × 3	1 (10%)	[21]
**Data augmentation techniques**	8 (100%)	
Rotation, flipping, zooming, etc.	4 (50%)	[21,26,34,40]
SMOTE-ENN	1 (13%)	[27]
GANs	1 (13%)	[28]
Others	2 (25%)	[31,39]
**Filtering techniques**	3 (100%)	
Guided filter-based denoising	1 (33%)	[34]
Bilateral filter	1 (33%)	[27]
Quantum matched-filter	1 (33%)	[35]
**Dimensionality reduction (PCA)**	2 (100%)	[35,36]
**Other techniques**	3 (100%)	[25,37,38]

Data standardization through image resizing was the most frequently applied preprocessing technique in the reviewed studies. This step is crucial for ensuring input data compatibility with CNN architectures and maintaining consistency across training and validation phases. The most commonly used input dimension was 224 × 224 × 3, reported in 3 studies (30%) [30,32,33], which aligns with the standard input size for widely used pretrained models such as VGGNet and ResNet. Other resolutions included 128 × 128 × 3, used in 2 studies (20%) [26,28], and several less conventional sizes that each appeared in 1 study (10% each): 176 × 176 × 3 [27], 100 × 100 × 3 [29], 112 × 112 × 3 [31], 227 × 227 × 3 [17], and 229 × 229 × 3 [21]. This variation in input dimensions reflects differences in model architectures, computational constraints, and dataset characteristics among the studies that incorporated image resizing in their preprocessing pipelines.

Data augmentation techniques were employed in 8 studies to address class imbalance and enhance model generalization. Conventional augmentation strategies such as rotation, flipping, and zooming were implemented in 4 of these studies [21,26,34,40]. In addition to these traditional methods, several studies incorporated more advanced augmentation techniques. For example, GANs were used to synthesize new training samples [28], while the SMOTE-ENN method—a hybrid technique combining synthetic minority oversampling and edited nearest neighbors—was applied to simultaneously oversample and clean the dataset [27]. Other context-specific strategies tailored to the characteristics of the AD datasets were also explored [31,39], aiming to enrich the representation of underrepresented classes and improve the robustness of the learning process.

Filtering methods were adopted in 3 studies with the goal of reducing noise and improving image quality, thereby enabling more accurate and reliable feature extraction. The techniques varied across studies and included guided filter-based denoising for edge-preserving smoothing [34], bilateral filtering to retain structural integrity while suppressing noise [27], and quantum matched filtering [35], which leverages principles from quantum computation to enhance image clarity. Dimensionality reduction techniques were applied in 2 studies to address the challenge of high-dimensional feature spaces commonly produced by CNNs. PCA was the method of choice, used to reduce computational complexity and improve model efficiency by projecting high-dimensional features onto a lower-dimensional subspace while preserving the most informative components [35,36].

Finally, a category labeled “Others” included diverse neuroimaging preprocessing strategies reported in 3 studies. These involved deskulling to remove nonbrain tissues; tissue segmentation to differentiate gray matter, white matter, and cerebrospinal fluid; and template matching to align MRI scans with standardized anatomical references. Such techniques are particularly important for enhancing anatomical accuracy and ensuring clinical relevance in brain imaging tasks [25,37,38].

### DL models used in the surveyed studies

In recent years, computer-aided diagnostic systems have become increasingly vital in disease detection and classification, largely due to advancements in DL and medical imaging. Among various DL architectures, CNNs have gained prominence for their ability to automatically learn hierarchical features from raw input data, eliminating the need for manual feature engineering typically required in traditional ML approaches.

Table 3 summarizes the DL models employed across the reviewed studies. Notably, 12 out of the 18 papers (67%) adopted pretrained or TL models [17,21,25–28,30–33,36,40]. These models, initially trained on large-scale datasets such as ImageNet, were fine-tuned for specific tasks, including AD classification.

**Table 3.** Deep learning models used in the surveyed papers

Deep learning models	No. of studies	References
Pre-trained/TL models	12 (67%)	[17,21,25–28,30–33,36,40]
ResNet (all variants)	5	[25,26,30,31,36]
Inception (V2, V4)	3	[21,27,31]
AlexNet	2	[17,36]
EfficientNet (B5, B7)	2	[26,32]
VGG	1	[26]
Xception	1	[26]
DenseNet	1	[26]
GoogleNet	1	[36]
**Custom CNN models**	6 (33%)	[29,34,35,37–39]

Among the TL models, ResNet and its variants were the most frequently utilized, appearing in 5 studies [25,26,30,31,36]. For example, Islam and Zhang [31] employed a ResNet-based architecture for feature extraction and classification, while Aparna and Rao [30] used ResNet50v2 to classify AD stages via optimized deep neural networks. Sisodia et al. [26] evaluated ResNet50 alongside other TL models, and Kumar and Sasikala [36] compared ResNet18, ResNet50, and ResNet101, combining them with ML classifiers such as SVM, KNN, Naive Bayes, and Decision Tree. Puente-Castro et al. [25] used a ResNet-based artificial neural network in conjunction with an SVM-RBF classifier.

Inception and its variants were used in 3 studies [21,27,31]. Rana et al. [27] proposed a hybrid model combining InceptionV2 with a custom CNN, while Islam and Zhang [21,31] employed InceptionV4.

AlexNet and EfficientNet models each appeared in 2 studies, [17,36] and [26,32], respectively. In both [17,36], AlexNet was used for feature extraction followed by ML classifiers. Sisodia et al. [26] used EfficientNetB7 in a comparative study, while Lokesh et al. [32] built a model named AzNet based on EfficientNetB5.

Additional TL models included VGG, Xception, and DenseNet—all used in Ref. [26] for class-wise AD prediction using MRI data—and GoogleNet, used in Ref. [36] for feature extraction and comparison with other TL models.

On the other hand, 6 studies (33%) utilized custom or nontransfer DL models [29,34,35,37–39]. Anitha et al. [34] employed a SegNet-based segmentation model with Restricted Boltzmann Machines. Amini et al. [35] and Bringas et al. [37] developed custom CNNs with hyperparameter tuning for fMRI-based AD classification. Mandal and Mahto [29] implemented a multibranch CNN architecture, while Jiang [38] proposed a sparse noise-reduction autoencoder with a softmax classifier.

### Average performances of the models in the covered studies

The DL models reviewed in this study were categorized into 2 groups: pretrained or TL models and custom-designed CNNs. Table 4 summarizes the average performance metrics reported for both groups across commonly used evaluation criteria, including accuracy, precision, recall (or sensitivity), specificity, F1 score, area under the receiver operating characteristic curve (AUROC), and area under the precision-recall curve (AUPRC).

**Table 4.** Average performance metrics of deep learning models in the surveyed papers

DL model type	Performance metric	Average (%)	References
Pre-trained/TL models	Accuracy	94.33	[17,21,25–28,30,32,36]
	Precision	80.47	[25–28,31,32,36]
	Recall/Sensitivity	79.39	[25–28,31,32,36]
	F1 score	79.23	[25–28,31,32,36]
	Specificity	95.06	[25,28,36]
	AUROC	99.88	[26,28]
Custom/Non-TL models	Accuracy	95.22	[29,34,35,37,38]
	Recall/Sensitivity	92.86	[34,35,37,38]
	Precision	94.78	[34,35,37]
	Specificity	96.01	[34,39]
	F1 score	93.09	[34,39]
	AUROC	81.00	[39]
	AUPRC	40.00	[39]

Among the studies using pretrained or TL models, accuracy was the most widely reported metric, appearing in 9 out of 12 papers. These models achieved an average classification accuracy of 94.33% [17,21,25–28,30,32,36]. Precision, recall (sensitivity), and F1 score were each reported in 7 studies [25–28,31,32,36], with corresponding average values of 80.47%, 79.39%, and 79.23%. Specificity was reported in 3 studies [25,28,36], with an average of 95.06%. AUROC was reported in 2 studies [26,28], yielding an average of 99.88%.

Custom DL models—those developed without leveraging pretrained weights—were evaluated using similar metrics. Accuracy was reported in 5 out of 6 relevant studies, averaging 95.22% [29,34,35,37,38]. Recall (sensitivity) and precision were reported in 4 and 3 studies, respectively [34,35,37,38], with average scores of 92.86% and 94.78%. F1 score and specificity were each reported in 2 studies [34,39], with averages of 93.09% and 96.01%, respectively. Additionally, AUROC and AUPRC were uniquely reported in Ref. [39], yielding averages of 81.00% and 40.00%, respectively, highlighting the use of threshold-independent performance metrics in more recent studies.

### Comparative synthesis and meta-analytic observations

This subsection moves beyond the descriptive summaries presented above to draw comparative and meta-analytic insights across the included studies. While the “Databases and data modalities” up to the “Average performances of the models in the covered studies” sections detailed data sources, preprocessing strategies, model families, and performance metrics individually, here we synthesize these elements into 3 overarching patterns. Specifically, we compare TL models versus custom CNN models in terms of headline accuracy (the “DL models used in the surveyed studies” and “Average performances of the models in the covered studies” sections), examine how differences in metric choice and task framing shape reported performance (the “Databases and data modalities” and “Average performances of the models in the covered studies” sections), and evaluate how dataset provenance and split granularity affect robustness (the “Databases and data modalities” and “Preprocessing techniques employed in the included studies” sections).

Beyond the descriptive averages in Table 4, 3 comparative patterns emerge. First, pretrained or TL backbones and CNNs, or non-TL models, attain similar headline performance at the level of the included literature. From the synthesis of the 18 papers in this scoping review, as also indicated in Table 4, TL studies report a mean accuracy of 94.33% (*N* = 9), while custom CNNs average 95.22% (*N* = 5)—a difference of less than one percentage point that does not indicate a dominant paradigm in aggregate. This pattern holds across the reviewed studies despite variability in architectural families and preprocessing pipelines (Table 4).

Second, performance estimates are highly sensitive to metric choice and task framing. With one exception, all included studies implemented 4-class staging designs (e.g., nondemented, very mild, mild, and moderate dementia). The sole exception is Mandal and Mahto [29], who excluded the moderate dementia category (*n* = 64) due to its small sample size and trained a 3-class model (nondemented, very mild, and mild). While this pragmatic choice mitigated class imbalance, it also highlights how dataset curation and class definition can influence apparent performance; their model reported an accuracy of 99.05%, higher than most 4-class articles in this review. More broadly, cross-sectional staging studies frequently report very high accuracies (often *≥*95%), whereas progression prediction tasks evaluated with time-to-conversion endpoints show markedly lower discrimination, with AUROC values of 0.80 to 0.82 (internal) and 0.75 to 0.83 (external), and corresponding AUPRC values of 0.27 to 0.46 [39]. Such contrasts underscore how class granularity, class balance, and endpoint definition materially alter baseline difficulty and apparent model success. Consequently, simple pooling of raw accuracies across heterogeneous multiclass designs obscures these nuances; median (interquartile range) summaries stratified by both metric (accuracy, AUROC, and AUPRC) and task framing (cross sectional vs. progression) provide a more faithful meta-analytic representation than single mean values. From a translational perspective, this inconsistency in reporting standards complicates benchmarking and impedes clinical integration, where robust and comparable performance metrics are essential.

Third, dataset provenance and split granularity are strongly associated with robustness. In our extraction, the most common primary source is ADNI alone (6/18; 33.33%), followed by OASIS (3/18; 16.67%), Kaggle (3/18; 16.67%), and smaller mixed combinations: Kaggle + ADNI (2/18; 11.11%), private datasets (2/18; 11.11%), OASIS + ADNI (1/18; 5.56%), and Kaggle + private (1/18; 5.56%). Studies relying on curated Kaggle or OASIS subsets frequently report near-ceiling internal accuracies (often *≥*97%) while acknowledging limited clinical readiness and the need to reconstitute pipelines on ADNI or other multisite datasets [29]. Moreover, stratified random subsampling at the image level—common in these works—risks information leakage when multiple scans per subject exist; subject-level splits and external site validation are essential to mitigate optimistic bias. Notably, only a single study in this scoping review explicitly implements external validation [39], highlighting a key translational gap.

Taken together, these comparative patterns illustrate that while reported accuracies appear consistently high, deeper synthesis reveals methodological heterogeneity, inconsistent evaluation practices, and limited external validation—all of which temper headline results and underscore the translational barriers that must be addressed for clinical deployment.

## Limitations and Future Directions Mentioned in the Covered Studies

### Limitations highlighted in the covered studies

This review identified several recurring limitations in the current literature on DL-based AD diagnosis and classification, which reveal important methodological gaps and practical constraints.

One of the limitations reported was the lack of biological variables and real-world clinical data, which restricted the ability of models to generalize to real-world settings. For example, definitions of rapid progression often relied solely on clinical scores, omitting multimodal indicators such as biomarker data or imaging-based thresholds that could improve diagnostic accuracy [39].

Dataset-related issues were also reported as a limitation in the reviewed papers. Many studies, including those by Ma et al. [39] and Rana et al. [27], relied on small and demographically homogeneous datasets, reducing model robustness and external validity. Moreover, heterogeneity in imaging protocols, resolutions, and labeling standards further hindered reproducibility and interstudy comparability [29].

The high computational cost of DL models was another significant concern. Training complex architectures often required substantial computational resources, especially when coupled with hyperparameter tuning and cross-validation [27,36]. This may limit model deployment in low-resource clinical settings.

Another limitation involved the scarcity of annotated neuroimaging data. Manual annotation is time-intensive and requires clinical expertise, restricting the scalability of supervised learning. Some studies suggested that semisupervised, unsupervised, and advanced data augmentation techniques may help alleviate this challenge [29,36].

Generalization to unseen datasets and handling of complex diagnostic decisions were also flagged as weaknesses, often attributed to overfitting, model bias, and insufficient training diversity [33]. Additionally, underutilization of MRI modalities or planes (e.g., sagittal and frontal) was noted to limit the depth of pathological insights [29].

Clinical relevance was also a concern. Few models addressed early-stage subtype prediction or explored causal mechanisms underlying AD progression, reducing clinical interpretability and decision support [40]. Furthermore, TL approaches were sometimes used without domain-specific fine-tuning, which may reduce real-world applicability [26].

In summary, these limitations emphasize the need for more diverse and multimodal datasets, scalable and efficient architectures, and clinically interpretable models to ensure the development of reliable and impactful diagnostic tools.

### Future directions highlighted in the covered studies

The reviewed studies proposed several promising directions to enhance the effectiveness and applicability of DL models in AD diagnosis.

One of the recommendations mentioned in the analyzed papers was the integration of multimodal clinical and biological data—such as electronic medical records (EMRs), genetic markers, and survival models—for more robust and personalized predictions [26,39]. This integration is expected to enhance model generalizability and real-world performance.

Computational scalability and ethical deployment were also emphasized. Rana et al. [27] advocated for testing models on larger and more diverse datasets using advanced computing resources. They also called attention to data privacy and security, urging researchers to consider these ethical challenges in real-world applications.

Improving data diversity and addressing class imbalance were frequently discussed. Kumar and Sasikala [36] suggested employing generative models, such as GANs and neural diffusion models, to synthetically augment minority classes and improve classification outcomes.

From an architectural perspective, Mandal and Mahto [29] proposed neural expert systems with parallel branches for different MRI modalities, potentially combined with federated learning for privacy-preserving distributed training. Bringas et al. [37] envisioned deploying such systems via cloud infrastructure, enabling continuous, remote AD monitoring using wearable sensors.

Expanding the scope of datasets and disease types was another forward-looking suggestion. Islam and Zhang [21] recommended testing models across multiple AD datasets and other neurological disorders to evaluate cross-condition performance.

Additional directions included leveraging underutilized MRI planes (e.g., sagittal and frontal) to enrich spatial information [25], refining discriminative features for improved TL [33], and developing user-friendly remote interfaces to support early detection and clinical decision-making [32].

Lastly, enhancing subtype classification and investigating causal pathways were seen as critical for advancing personalized treatment and understanding of AD pathophysiology [40].

Collectively, these directions advocate for interdisciplinary collaboration, advanced modeling frameworks, and patient-centered solutions to bridge current gaps and translate DL advancements into clinically actionable outcomes.

### Clinical translation: Generalizability, workflow integration, and cost-effectiveness

Clinical translation of DL models for AD requires moving beyond benchmark performance to demonstrate robustness across diverse clinical settings. In this scoping review, several included studies note that reliance on single-source datasets constrains generalizability and highlight the need for multisite validation as a minimum translational standard [27,29,39]. For example, Mandal and Mahto [29] report the necessity of adapting preprocessing pipelines when shifting from Kaggle to ADNI or OASIS, while Ma et al. [39] show that trial-based models face challenges when applied to routine EMRs due to batch effects and inclusion/exclusion biases. Collectively, these findings underscore that subject-level data splits, external hold-outs, and consistent reporting of AUROC and AUPRC are essential to establish credible generalization.

From a workflow perspective, practical deployment requires end-to-end pipelines that integrate seamlessly into radiological practice. Across the reviewed studies, key steps were reported such as Digital Imaging and Communications in Medicine cleaning, image conversion, intensity normalization, and disciplined train/test segregation [25,28]. These preprocessing routines, when coupled with structured inference outputs—such as Gradient-weighted Class Activation Mapping or attention overlays—provide clinicians with interpretable results that can be incorporated into picture archiving and communication systems. Establishing such standardized workflows, with radiologist-in-the-loop adjudication, represents a critical step toward real-world implementation.

Cost-effectiveness, though rarely addressed explicitly, emerges indirectly from the included papers. Mandal and Mahto [29] emphasize that reliance on freely available datasets such as Kaggle reduces financial barriers to replication. Similarly, studies using ADNI and OASIS not only acknowledge the advantage of open access but also recognize the resource demands of adapting these pipelines for multisite or trial-derived data [27,39]. Ma et al. [39] further note that scaling trial-based models into EMR workflows requires substantial reengineering, with associated financial costs in harmonization and preprocessing. Taken together, these findings suggest that cost-effectiveness in DL for AD is tied less to algorithmic efficiency than to data accessibility and clinical integration. Future studies should make financial considerations explicit by reporting costs of data acquisition, preprocessing, and deployment, enabling more realistic assessments of feasibility in healthcare settings.

In summary, effective clinical translation of DL models for AD requires more than achieving high accuracy—it demands robust generalization across sites, seamless integration within clinical workflows, and transparent consideration of financial costs. Addressing these dimensions collectively will be essential for transforming current experimental models into reliable, scalable tools for real-world healthcare practice.

## Conclusion

This scoping review shows that DL models achieve strong performance in AD stage classification but face barriers to generalizability, workflow integration, and clinical adoption. Most models rely on limited datasets, lack external validation, and often underreport complexity or deployment requirements. By aligning technical innovation with clinical requirements, the field can progress closer to realizing the full potential of DL in early diagnosis, disease monitoring, and personalized intervention strategies for AD.

Our comparative synthesis further indicates that TL and custom CNNs yield nearly indistinguishable headline performance, suggesting no dominant paradigm at present. Reported outcomes vary substantially depending on task framing and dataset provenance, and only one study included external validation, leaving robustness largely untested. Moreover, financial cost-effectiveness remains underexplored, though several studies highlight the tension between freely available datasets that lower entry barriers and the significant resource demands of adapting pipelines for multisite or EMR data. Addressing these gaps—particularly external validation, reporting of financial costs, and workflow integration—will be essential to ensure clinically meaningful translation.
